# Intracellular Trafficking of Cationic Carbon Dots in Cancer Cell Lines MCF-7 and HeLa—Time Lapse Microscopy, Concentration-Dependent Uptake, Viability, DNA Damage, and Cell Cycle Profile

**DOI:** 10.3390/ijms23031077

**Published:** 2022-01-19

**Authors:** Markéta Havrdová, Iztok Urbančič, Kateřina Bartoň Tománková, Lukáš Malina, Kateřina Poláková, Janez Štrancar, Athanasios B. Bourlinos

**Affiliations:** 1 Regional Centre of Advanced Technologies and Materials, Czech Advanced Technology and Research Institute (CATRIN), Palacký University Olomouc, Křížkovského 511/8, 779 00 Olomouc, Czech Republic; katerina.polakova@upol.cz; 2 Laboratory of Biophysics, Condensed Matter Physics Department, Jožef Stefan Institute, Jamova Cesta 39, 1000 Ljubljana, Slovenia; iztok.urbancic@ijs.si (I.U.); janez.strancar@ijs.si (J.Š.); 3 Department of Medical Biophysics, Faculty of Medicine and Dentistry, Institute of Translational Medicine, Palacký University in Olomouc, Hněvotínská 3, 775 15 Olomouc, Czech Republic; katerina.barton@upol.cz (K.B.T.); lukas.malina@upol.cz (L.M.); 4 Physics Department, University of Ioannina, 45110 Ioannina, Greece; bourlino@uoi.gr

**Keywords:** cationic carbon dots, fluorescence microspectroscopy, nucleus, cytotoxicity, genotoxicity, cancer cells, MCF-7, HeLa

## Abstract

Fluorescent carbon dots (CDs) are potential tools for the labeling of cells with many advantages such as photostability, multicolor emission, small size, rapid uptake, biocompatibility, and easy preparation. Affinity towards organelles can be influenced by the surface properties of CDs which affect the interaction with the cell and cytoplasmic distribution. Organelle targeting by carbon dots is promising for anticancer treatment; thus, intracellular trafficking and cytotoxicity of cationic CDs was investigated. Based on our previous study, we used quaternized carbon dots (QCDs) for treatment and monitoring the behavior of two human cancer cell MCF-7 and HeLa lines. We found similarities between human cancer cells and mouse fibroblasts in the case of QCDs uptake. Time lapse microscopy of QCDs-labeled MCF-7 cells showed that cells are dying during the first two hours, faster at lower doses than at higher ones. QCDs at a concentration of 100 µg/mL entered into the nucleus before cellular death; however, at a dose of 200 µg/mL, blebbing of the cellular membrane occurred, with a subsequent penetration of QCDs into the nuclear area. In the case of HeLa cells, the dose-depended effect did not happen; however, the labeled cells were also dying in mitosis and genotoxicity occurred nearly at all doses. Moreover, contrasted intracellular compartments, probably mitochondria, were obvious after 24 h incubation with 100 µg/mL of QCDs. The levels of reactive oxygen species (ROS) slightly increased after 24 h, depending on the concentration, thus the genotoxicity was likely evoked by the nanomaterial. A decrease in viability did not reach IC 50 as the DNA damage was probably partly repaired in the prolonged G0/G1 phase of the cell cycle. Thus, the defects in the G2/M phase may have allowed a damaged cell to enter mitosis and undergo apoptosis. The anticancer effect in both cell lines was manifested mainly through genotoxicity.

## 1. Introduction

Over recent decades, the carbon dots (CDs) have been very popular as promising fluorescent probes due to their low photobleaching, lack of optical blinking, tunable photoluminescence, versatile surfaces, and excellent biocompatibility [1,2]. These excellent properties of CDs have made them prosperous in the applications of bioimaging, drug delivery, biochemical detection, and sensors [3,4,5,6,7]. Passivation of nanoparticles including CDs is a very commonly used procedure which equips the nanomaterials with different properties such as charge [8,9], fluorescence, surface specificity, and affinity to the cellular structures (cellular membrane, organelles, proteins, genes) [9] and influent cellular uptake [8,10], drug delivery [11,12,13], gene transfection efficacy [14,15,16,17], and general biological effects [18]. Synthesis of cationic carbon dots can be achieved by a facile ultrasonic method [19], green melting method [20], hydrothermal method [21,22], thermal oxidation [23,24,25]. Positively charged carbon dots are promising for in vitro applications (genetic engineering [26], biosensors [27], diagnosis [28], antibacterial agents [25]). They can show different behaviors in various cell lines, as we already studied with regard to healthy animal cell lines [8,29,30]. A variety of CDs have been applied for labeling different subcellular structures [31]; however, most CDs mainly accumulate in the cytoplasm, especially in endo/lysosomes, mitochondria, Golgi apparatus, and endoplasmic reticulum [32]. CDs have been studied also for nucleus labeling, photodynamic therapy, and optical monitoring of anticancer drugs [33,34,35,36]. DNA-damage can be caused by the small size of nanoparticles together with their surface charge and surface functional groups, which alters the interaction of CDs with cells [37]. Since many anticancer drugs are required to enter the cell nucleus where the drugs damage the genes to stop the proliferation of the cancer cell [28,38,39], nucleus targeting, optical monitoring, genotoxic effect, inhibition of proliferation, and targeting of mitochondria are all regarded as crucial properties of nanomaterials for anticancer treatment. Thus, the present work is focused on QCDs trafficking and cytotoxic assays to obtain a comprehensive study for future anticancer drug delivery complexes.

## 2. Results

### 2.1. Intracellular Observation of QCDs-Labeled MCF-7 Cells

**Figure 1:**
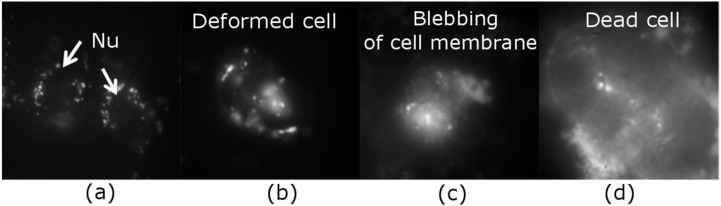
MCF-7 cells treated with concentrations of 50–400 µg/mL of QCDs for 24 h: (**a**) 50 µg/mL: contrasted perinuclear area of two cells, “Nu” denotes nucleus area; (**b**) altered morphology of cell (ring-shape) caused by a concentration of 100 µg/mL; (**c**) dying cell after incubation with 200 µg/mL—blebbing of cell membrane; (**d**) cluster of dead cells after exposure to 400 µg/mL; FMS, the field of view was 91 µm in images (**a**–**c**), and 55 µm in image (**d**).

**Figure 2:**
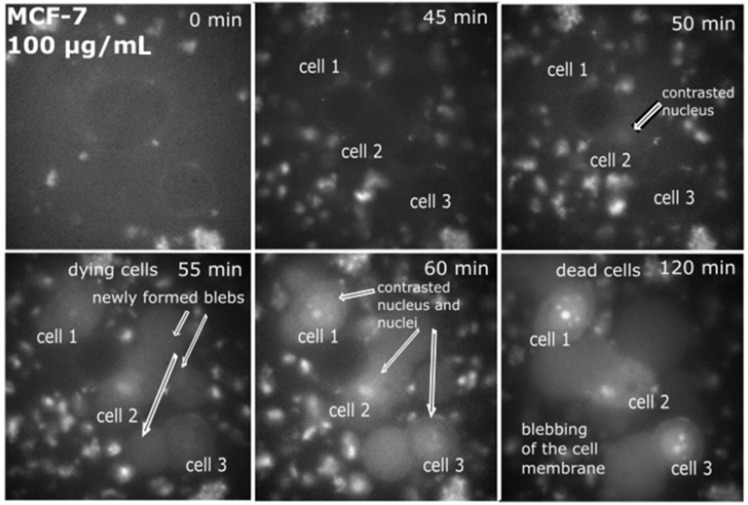
Live monitoring of MCF-7-labeled cells observed in growth medium; time 0 min = time immediately after addition of the solution with a concentration of 100 µg/mL of QCDs in the growth medium; at time 50 min, it is obvious that the first nucleus was contrasted, after next 5 min (at time 55 min), all the cells started blebbing, indicating the first sign of cellular death. FMS, total magnification 90, field of view 91 µm.

**Figure 3:**
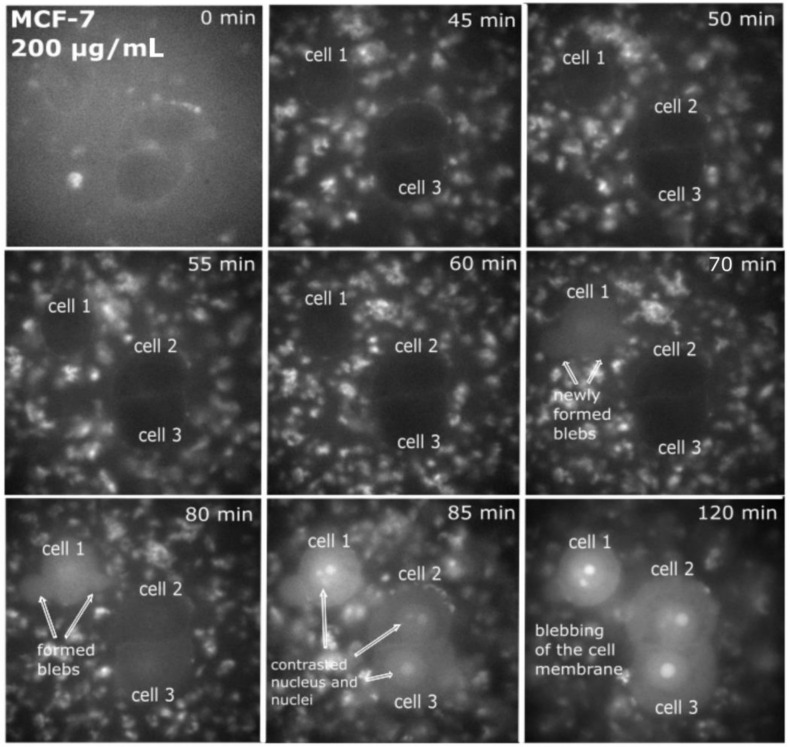
Live monitoring of MCF-7-labeled cells in growth medium; time 0 min = time immediately after addition of the solution with a concentration of 200 µg/mL of QCDs in the growth medium; first cell was contrasted at time 70 min and at this time, the first blebs also appeared; at 80 min, contrasted nucleus was obvious and was enhanced with time and also other nuclei and nucleoli were tagged (see 85 and 120 min). FMS, total magnification 90, field of view 91 µm.

**Figure 4:**
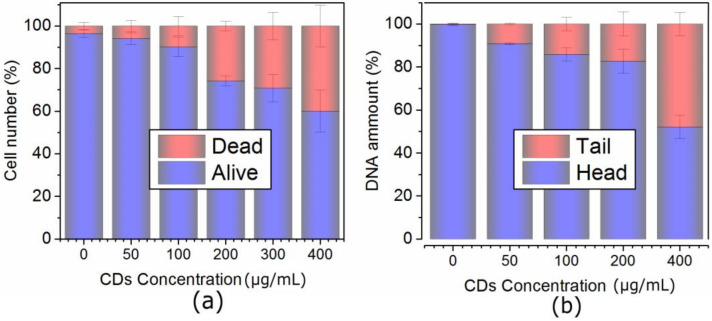
(**a**) Viability and (**b**) genotoxicity of MCF-7 cells exposed to the concentration line of QCDs after 24 h incubation. Please note genotoxicity terms: “head” is intact DNA in nucleus, “tail” is damaged DNA migrated away from the nucleus. When the tail value is more than 10%, the dose is considered genotoxic [29].

**Figure 5:**
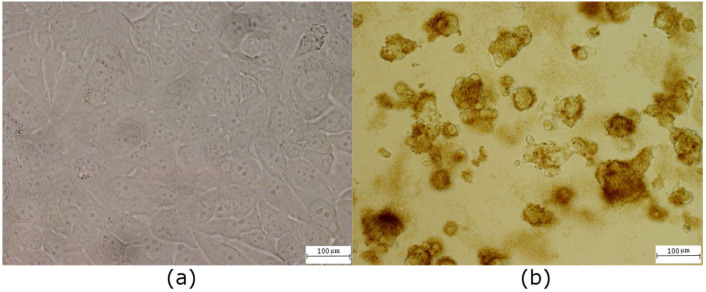
Light microscopy of MCF-7 cells: (**a**) non-labeled control cells, confluence 100%; (**b**) the same number of cells labeled with 200 µg/mL of QCDs and their anticancer effect after 24 h; scale bar 100 µm.

Cancer MCF-7 cell line was incubated with various doses of QCDs, i.e., 50, 100, 200, 400 µg/mL for 24 h, and imaged (Figure 1). MCF-7 cells treated with 50 µg/mL of QCDs had full endo/lysosomes in perinuclear area (Figure 1a). Concentration of QCDs from 100 µg/mL changed the cellular morphology to the ring-shape (Figure 1b–d); thus, it was not possible to recognize whether the QCDs were present in the nucleus or not. For better information on interaction of cells with a nanomaterial, live monitoring was recorded immediately after addition of QCDs. It was obvious that QCDs enter into the nuclei after 45 min incubation with cells (see Figure 2) and after 24 h, the cells revealed weak adherence and were dying, as in the case of L929 cells [29]. Weak adherence of labeled MCF-7 cells caused a decrease in a number of cells for imaging in higher doses. At a concentration of 200 µg/mL, we observed that the penetration into the nuclei occurred surprisingly later (between 65–70 min, see Video S1) than at a concentration of 100 µg/mL; however, the blebbing happened at the same time as the signal enriched the nuclei (Figure 3). Thus, according to microscopy evaluation, we know that the morphology of MCF-7 cells was significantly deformed at a dose of 100 µg/mL and that the presence of QCDs in the nuclei and nucleoli happened before cellular dying as blebbing of cellular membrane occurred after the QCDs contrasted the nuclear area of MCF-7 cells. Although, cells had weak adherence and viability was not reduced strongly, as witnessed from the cytotoxicity measurements (Figure 4a), we considered this dose as critical based on changes of morphology. The comet assay demonstrated a damage of DNA. A concentration of 50 µg/mL evoked a value of the tail less than 10% (please note: the “head” is intact DNA in the nucleus, the “tail” is damaged DNA migrated away from the nucleus; when the tail value is less than 10%, the dose is non-genotoxic). At a concentration of 100 µg/mL, the value of the tail was 14.11% (Figure 4b). Subsequently, genotoxicity was growing with the increasing concentration of QCDs and reached 50% at 400 µg/mL (see Figure 4b). In summary, very sensitive doses of QCDs for MCF-7 cells are 100 and 200 µg/mL. However, the faster killing, monitored during the first two hours immediately after adding of the sample, occurred at a concentration of 100 µg/mL. QCDs at higher concentrations covered the cells and restricted movement and proliferation. This is also viewed as a beneficial effect in antitumor treatment (Figure 5).

### 2.2. Intracellular Observation of QCDs-Labeled HeLa Cells

**Figure 6:**
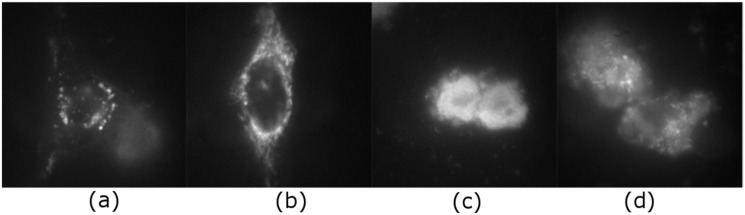
HeLa cells contrasted by 50–400 µg/mL of QCDs: (**a**) 50 µg/mL localization of endo/lysosomes filled by QCDs in perinuclear area; (**b**) QCD-contrasted mitochondria at a dose of 100 µg/mL; (**c**) 200 µg/mL—critical dose for HeLa cells—death mainly of mitotic cells; (**d**) 400 µg/mL—dead HeLa cells with blebs. FMS, total magnification 90, field of view 91 µm.

**Figure 7:**
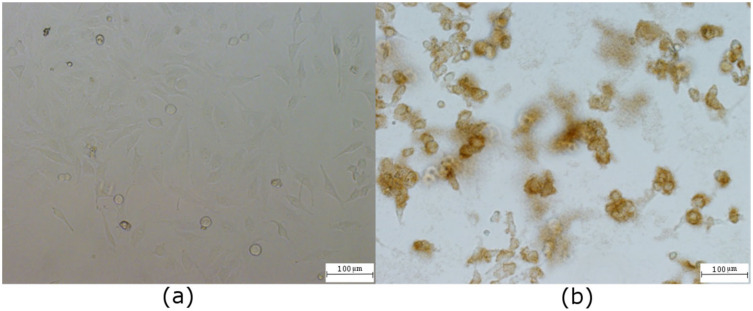
Light microscopy of HeLa cells: (**a**) non-labeled control cells, confluence 100%, (**b**) the same number of cells labeled with 200 µg/mL of QCDs and their anticancer effect after 24 h.

**Figure 8:**
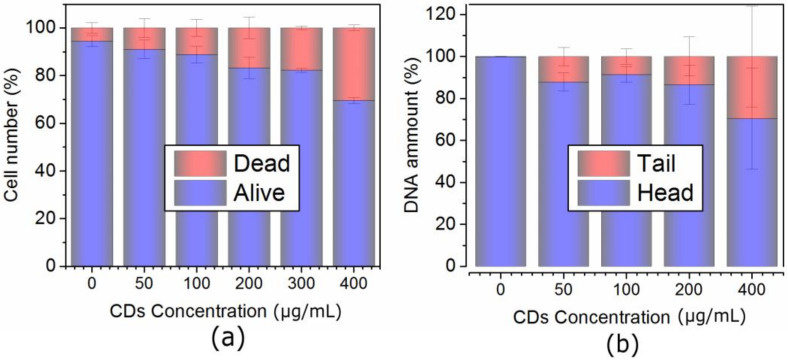
(**a**) Viability and (**b**) genotoxicity of HeLa cells exposed to the concentration line of QCDs after 24 h incubation. Please note genotoxicity terms: “head” is intact DNA in nucleus, “tail” is damaged DNA migrated away from the nucleus. When the tail value is more than 10%, the dose is genotoxic [29].

The same concentrations, conditions, and techniques were also used in the case of HeLa cells. At a concentration of 50 g/mL, QCDs were located in cytosol and around the nuclei in endo/lysosomes (Figure 6a); however, 100 µg/mL of QCDs filled the whole cytosol and probably interacted with mitochondria (see Figure 6b). Mitochondria in human cancer cells are closely related to cancer cell proliferation, invasion, metastasis, and drug-resistant mechanisms, making them promising target organelle for the anticancer treatment [40]. Carbon dots, from a concentration of 200 µg/mL, entered into the nuclei (confirmed by live monitoring—QCDs entered in nucleus after 75 min, see Video S2) and after 24 h, they caused cellular death especially in mitotic cells (Figure 6c and Figure 7). Those cells which survived the highest dose, i.e., 400 µg/mL, of QCDs were massively deformed and detached, since after washing, the resulting number of cells was very low (Figure 6d). It seems that anticancer dose of QCDs for HeLa cells occurred at a concentration of 200 µg/mL. Nevertheless, viability (Figure 8a) was gradually decreased at all the doses. To check the mechanisms behind different viability, and considering strong partitioning of QCDs into nucleus, we compared genotoxicity (Figure 8b).

Genotoxicity in the HeLa cells occurred nearly at all the doses (12.13% at 50 µg/mL, 8.54% at 100 µg/mL, 13.42% at 200 µg/mL, and 29.56% at 400 µg/mL)—Figure 8b. Despite the fact that QCDs enter into the nuclei and nucleoli, as also seen in our previously tested healthy cell lines NIH/3T3 and L929 [29], we can assume that they caused genotoxicity only in cancer MCF-7 and HeLa cells. Until now, only a few studies have focused on genotoxicity caused by carbon dots themselves [30,41]; CDs are usually examined as biosensors for DNA detection [42,43,44,45].

### 2.3. Concentration-Dependent Uptake and Oxidative Stress in Both Cell Lines

**Figure 9:**
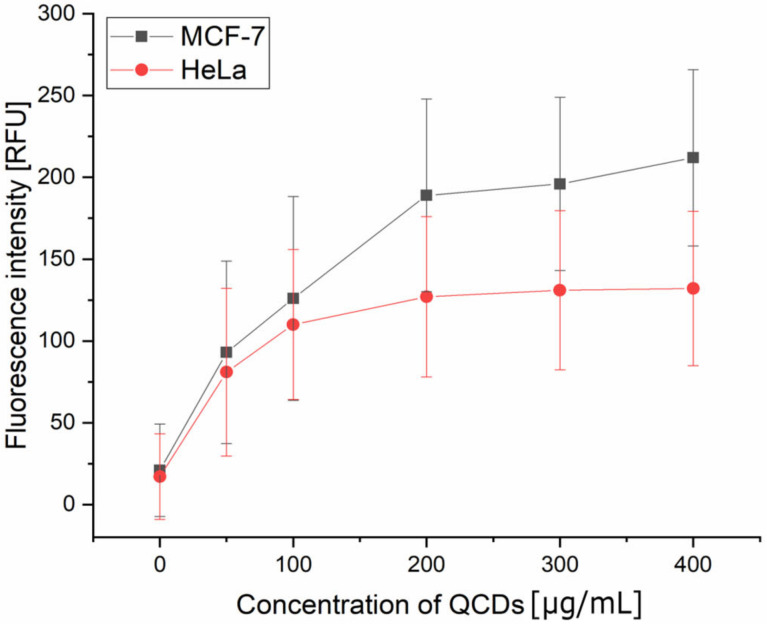
Concentration-dependent uptake of MCF-7 and HeLa cells, measured by flow cytometer. It needs to be mentioned that, for error bars in flow cytometry of concentration uptake measurement, values of coefficient of variations for events in each sample were used.

Intracellular trafficking and cytotoxicity depend also on the surface properties of the sample and on the number of incorporated nanomaterials into the cells as it can disrupt cellular homeostasis [46]. From our microscopic results, especially on the MCF-7 cell line, it was obvious that the concentration of QCDs in the interval of 100–200 µg/mL evoked different contrasting and cellular death. Thus, we measured fluorescence intensity of QCDs in individual cells by flow cytometry and tried to reveal the cause of different cell sensitivity. Cells were incubated with QCDs at concentrations from 0 to 400 µg/mL for 24 h at 37 °C, then trypsinized and measured by flow cytometry (Figure 9). From the observed results, it is obvious that the uptake of both cell lines is similar at a concentration of 50 µg/mL. At a dose of 100 µg/mL, a high increase in the uptake of MCF-7 cells is observed in comparison to HeLa cells, for which the uptake occurs in a more gradual manner. This measurement confirmed the microscopic results because the highest uptake of MCF-7 cells is found between the concentrations of 100–200 µg/mL, and the saturated concentration of QCDs is 200 µg/mL in the case of the HeLa cells. 

This assay was measured in parallel with mouse NIH/3T3 and L929 fibroblasts and we found out interesting similarities between NIH/3T3 and HeLa cells vs. L929 and MCF-7 cells (see Figure S1). The main changes occurred at 100 µg/mL, when similarity of uptake can be strongly visible in two groups (NIH/3T3 and HeLa cells vs. L929 and MCF-7 cells) and persisted up to the highest doses. These results showed that L929 and MCF-7 cells have a significantly stronger uptake than NIH/3T3 and HeLa cells. Quantitative determination of internalized CQDs in different cell lines was also described in the study [7] focused on the concentration-dependent photoluminescence of nitrogen-containing carbonaceous quantum dots (N-CQDs). Moreover, the authors in another study analyzed oxidative stress conditions by examining the cellular anti-oxidative capacity as a defensive response to the increasing concentration of N-CQDs [47].

**Figure 10:**
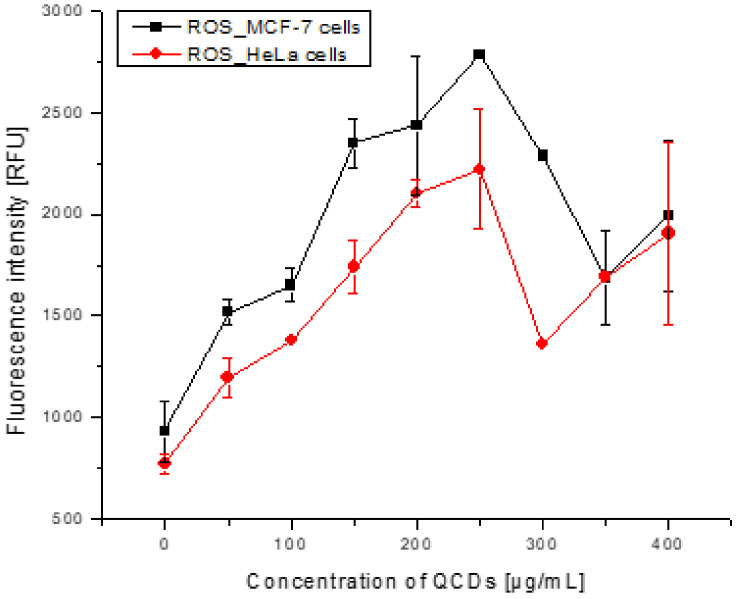
ROS level in MCF-7 and HeLa cells after 24 h incubation with different doses of QCDs.

Cellular uptake of the nanoparticles relates to the induction of intracellular reactive oxygen species (ROS) [48]. A low ROS level is generated by normal cell metabolism (physiological oxidative stress) whereas a high ROS production leads to oxidative damage of cells and death caused by excessive and toxic oxidative burden [49]. Thus, the oxidation stress of both cell lines was tested after 24 h incubation with QCDs (Figure 10). The ROS level was not significant up to 100 µg/mL and although subsequently ROS increased up to a concentration of 250 µg/mL, the value of the ROS level was not high in comparison to other studies [8,50]. Therefore, the ROS production was not identified as the major mechanism of cell damage. The oxidative stress may also lead to the genomic instability [51], but according to these results, the genotoxicity was likely evoked by the nanomaterial.

Cellular damage depends on the repairing of DNA in the cell cycle, where only cells with intact DNA can continue to mitosis and cells with damaged DNA undergo cellular death [52]. Thus, the cell cycle profile was measured.

### 2.4. Cell Cycle Analysis of MCF-7 and HeLa Cells

**Figure 11:**
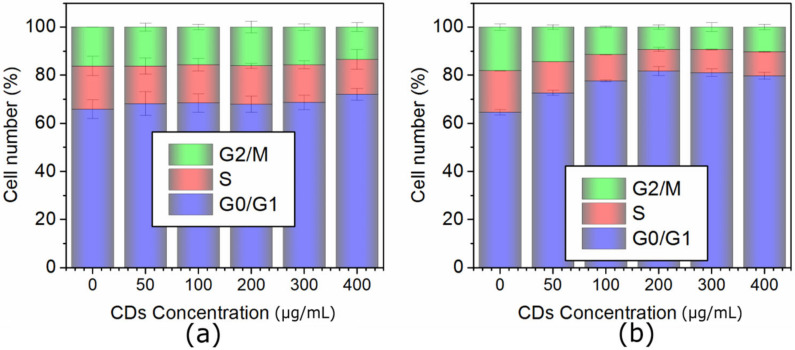
Cell cycle profile of cells labeled with QCDs: (**a**) human breast cancer MCF-7 cells, (**b**) human cervix cancer HeLa cells.

The cell cycle profile was analyzed by the flow cytometer using the DNA kit (BD CycletestTM Plus DNA kit, East Rutherford, NJ, USA) as in our previous work [29]. The cell cycle of MCF-7 labeled by QCDs was found to show no significant changes at the concentrations of 50–300 µg/mL. Nevertheless, in comparison to the highest dose (400 µg/mL) with the control non-labeled cells, the G0/G1 phase was slightly prolonged and the G2/M phase shortened (Figure 11a). Defects in the G2/M phase may allow a damaged cell to enter mitosis and undergo apoptosis [53]. In the case of QCDs treated-HeLa cells, all doses affected the cell cycle profile because, with growing concentration of QCDs, the G0/G1 phases were prolonged and G2/M phase shortened (Figure 11b). These changes can be a sign of low proliferation with increasing doses, of the entering of damaged cells in G0, and of the activation of apoptosis, or may be also a sign of the repairing of damaged DNA, which was confirmed in our genotoxicity measurement (Figure 8b).

## 3. Materials and Methods

This work was performed in parallel with our previous study [29]; thus, the method is very similar or the same.

### 3.1. Carbon Dots

**Figure 12:**
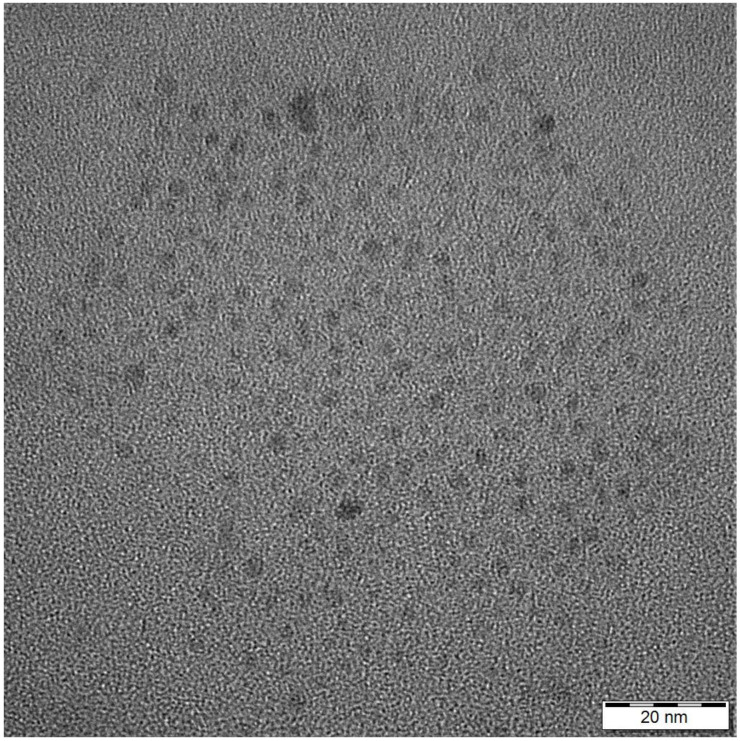
TEM image of QCDs, scale bare 20 nm.

Quaternized carbon dots (QCDs) were prepared by thermal oxidation of a tris(hydroxymethyl)aminomethane (Tris)—betaine hydrochloride salt precursor, where Tris provides the carbon source and betaine the surface modifier [23]. The QCDs have sizes in the range 4–9 nm, quasi-spherical morphology (Figure 12), and display a positive zeta potential of +43 mV at neutral pH (e.g., the pH of the QCDs’ aqueous dispersion). The presence of -N(CH_3_)^3+^ groups in QCDs was evidenced by NMR spectroscopy, as well as, by the highly positive zeta potential at neutral pH. Furthermore, based on elemental analysis and TGA, an anion-exchange capacity of 2.1 mmoL g^−1^ was estimated in the chloride form (Cl^−^ ions compensate the positively charged quaternary ammonium groups). Regarding the quantum yield of the dots (4%), this was estimated for the blue part of the emission spectrum, where PL had the highest intensity. As we moved to greater wavelengths the emission, and hence the quantum yield of the dots, was significantly decreasing. High quantum yields are usually considered suspicious in terms of purity of the dots and origin of fluorescence [54]. In the present case, the value of 4% is typical of carbon dots void of any fluorescent impurities. In general, temperature synthesis below 200 °C favors the formation of molecular fluorophores that dramatically increase the quantum yield. However, removal of such impurities by extensive purification results in quantum yields of 1–3%. On the other hand, temperature synthesis above 200 °C (as is true for our dots) results in higher degrees of carbonization and less fluorescent impurities formation with concurrent drop in quantum yield [55]. The purity of the dots used in this work was evidenced by capillary electrophoresis, where a narrow and single peak was noticed in the corresponding chromatograph. For more information on the material characterization, please see our previous studies [23,29].

### 3.2. Cell Cultivation, Microscopy

Both cell lines (MCF-7, HeLa) were purchased from American Type Culture Collection (ATCC, Manassas, VA, USA) and were cultivated in high glucose DMEM (Life Technologies, Carlsbad, CA, USA). Both media also contained 10% fetal calf serum (FCS), 10,000 U/mL penicillin, and 10,000 µg/mL streptomycin. Cells were incubated at 37 °C and under a 5% CO_2_-enriched atmosphere. A light microscope Olympus IX 70 equipped with a phase contrast was used for control of cell confluence.

### 3.3. Fluorescence Microspectroscopy

MCF-7 or HeLa cells were seeded on glass-bottom cell culture dishes (NuncTM, ThermoFisher Scientific, Waltham, MA, USA) at a cell density of 7 × 10^3^ and cultured for 24 h at 37 °C and 5% CO_2_. The next day, QCDs were diluted in the cell medium and added to each culture dish to achieve the desired final concentration (50, 100, 200, or 400 µg/mL), and left to incubate for 24 h. Before imaging, the dishes were washed twice with PBS and filled with a solution of HEPES and PBS (1:9). For live monitoring of the uptake, images were taken for 2 h in 5-min intervals immediately after the addition of QCDs (400 µg/mL). 

CDs were excited by widefield illumination with an Hg arc lamp (Sutter Lambda LS, Novato, CA, USA) through an excitation filter with the transmission window 430–490 nm, and emission was collected within 506–594 nm (all filters by Semrock, West Henrietta, NY, USA), using a 100×/1.4 oil immersion objective (Nikon, Tokyo, Japan). As reported previously [56], spectrally resolved images were acquired sequentially by scanning the transmission window of the liquid crystal tunable filter (Cri Varispec VIS-10-20, Cambridge Research & Instrumentation, Inc., Hopkinton, MA, USA), placed in front of an EMCCD camera (Andor iXon3 897, Oxford Instruments, Oxfordshire, UK), at 5-nm steps. From each 3D dataset (x,y,λ), spatially resolved emission spectra were extracted and analyzed using a custom spectral fitting software written in Wolfram Mathematica [57] to determine local intensities and spectral peak positions.

### 3.4. Cell Cycle and Concentration-Dependent Uptake

A BD FACSVerse flow cytometer (BD biosciences, East Rutherford, NJ, USA) was used for determination of cell cycle, concentration-dependent uptake, and endocytosis analysis. Cells were incubated 24 h with various concentration of QCDs and according to the BD protocol, DNA kit (BD CycletestTM Plus DNA kit, Becton Dickinson, East Rutherford, NJ, USA) was used. The fluorescent intensity of Propidium Iodide (PI) was measured using exc. 488 nm/ em. 586 nm detector to define any changes in the phases of the cell cycle.

Concentration-dependent uptake was assessed based on the mean fluorescent intensity (MFI) of labeled cells. We used concentrations of 50, 100, 200, 300, and 400 µg/mL of QCDs and after 24 h incubation, the supernatant was removed, and cells gently washed with PBS solution (0.1 M, pH 7.4). Subsequently, cells were detached with trypsin (0.25% in EDTA, Sigma-Aldrich, St. Louis, MO, USA), resuspended in 100 μL of growth medium, and fluorescence intensity of labeled cells was measured with a flow cytometer.

### 3.5. Viability

Viability of cells treated with QCDs was investigated by a BD FACSVerse flow cytometer (BD Biosciences, East Rutherford, NJ, USA) using LIVE/DEAD® Viability/Cytotoxicity Kit (Thermofisher, Waltham, MA, USA). Both cell lines (MCF-7 and HeLa) were labeled with the concentration line of 0–400 µg/mL of QCDs and incubated 24 h. Subsequently, cells were washed by PBS solution (0.1 M, pH 7.4), detached with trypsin (0.25% in EDTA, Sigma-Aldrich, St. Louis, MO, USA), and resuspended in 300 μL of growth medium. This LIVE/DEAD® Viability/Cytotoxicity Kit is the assay utilized to quantitate apoptotic cell death. Thus, cells were incubated with 2 µL of ethidium bromide (2 mM) a 2 µL of calcein-AM (50 µM), diluted in DMSO. The fluorescence signal was measured by flow cytometry (red—exc. 488/ em. 700, green—exc.488/em.527). Red signal of ethidium bromide marked dead cells because they lost membrane integrity. However, green cells had active intracellular esterases and catalyzed the non-fluorescent calcein-AM to highly fluorescent green calcein [29].

### 3.6. Comet Assay

Genotoxicity was studied by the comet assay which detects DNA damage. The principle of this method is based on single cell gel electrophoresis (SCGE), during which intact DNA stay in the nucleus (called “head”) and damaged DNA migrate away from the nucleus (resemble “tail” of comet). Specific DNA fluorescent probe allows to compare fluorescent intensity of the nucleoid (head) and migrated DNA (tail) in the image analysis [58]. In our study, we followed the methods from the article [59]. Microscope slides were covered with 1% HMP agarose, thereafter, the cells were trypsinized, washed with DMEM with 10% FBS, and centrifuged (6 min, 1000 rpm). Agarose (85 μL of 1% LMP) was added to the cell suspension and 85 μL of this mixture was given to the microscope with agarose gel. The microscope slides were immersed in a lysis buffer for 1 h, and then placed in an electrophoretic tank and dipped into a cool electrophoresis solution for 40 min. Electrophoresis was run at 0.8 V/cm and 380 mA for 20 min. Finally, slides were neutralized in buffer (0.4 M Tris, pH = 7.5) and the samples were stained with SYBR® Green and immediately scored using SW Comet Score [29]. 

### 3.7. Reactive Oxygen Species

Intracellular oxidative stress caused by QCDs was investigated by ROS analysis. At first, MCF-7 and HeLa cells were treated with 50–400 µg/mL of QCDs and incubated for 24 h. After incubation, the growth medium containing QCDs was removed and replaced by PBS solution (20 µL per well) containing fluorescence ROS probe (pre-dissolved in DMSO, 500 mmoL × L^−1^, General Oxidative Stress Indicator CM-H2DCFDA, Life Technologies) [8,60]. The fluorescence signal was measured by a microplate reader PRO M200 (Tecan, Austria) with excitation/emission wavelength of 505/529 nm [8].

## 4. Conclusions

In this work, we observed different sensitivity of two cancer cell lines to cationic carbon dots with an average size of 7 nm, and similarity between human cancer cells and mouse fibroblasts in the uptake of QCDs. Time lapse microscopy of QCDs-labeled MCF-7 cells showed that morphology changes and targeting of the nuclei occurred faster at a lower dose than at a higher one but just in case of the 2 h measurement, which was performed immediately after the addition of QCDs into the growth medium. Viability after 24 h did not change significantly at a concentration of 100 µg/mL, but the morphology of MCF-7 was still deformed. From the same concentration, genotoxicity was pronounced. In the case of HeLa cells, the dose-depended effect during the first 2 h did not happen; however, cells incubated with 200 µg/mL for 24 h were dying mainly in mitosis, probably because of weaker adherence. Genotoxicity occurred nearly at all the doses; moreover, contrasted subcellular compartments (probably mitochondria) were obvious after 24 h incubation with 100 µg/mL of QCDs. Knowledge of the intracellular fate of carbon dots is useful in a wide range of biological and biomedical applications. This sample deformed cellular shape, entering into the nucleus, causing mitotic catastrophe, and restricting movement and proliferation, which can all be viewed as beneficial effects in antitumor treatment.

## Data Availability

Not applicable.

## Supplementary Materials

The following supporting information can be downloaded at: https://www.mdpi.com/article/10.3390/ijms23031077/s1.
