# Supplementary material for: Intracellular Trafficking of Cationic Carbon Dots in Cancer Cell Lines MCF-7 and HeLa—Time Lapse Microscopy, Concentration-Dependent Uptake, Viability, DNA Damage, and Cell Cycle Profile
Source: Int J Mol Sci. 2022 Jan 19;23(3):1077. doi: 10.3390/ijms23031077 (PMC8835431; doi:10.3390/ijms23031077)
Supplement: Supplementary file 1 [file ijms-23-01077-s001.zip › SI.pdf]

Supplement Materials

Intracellular trafficking of cationic carbon dots in cancer cell lines MCF-7 and HeLa – Time lapse microscopy, concentration dependent uptake, viability, DNA-damage and cell cycle profile

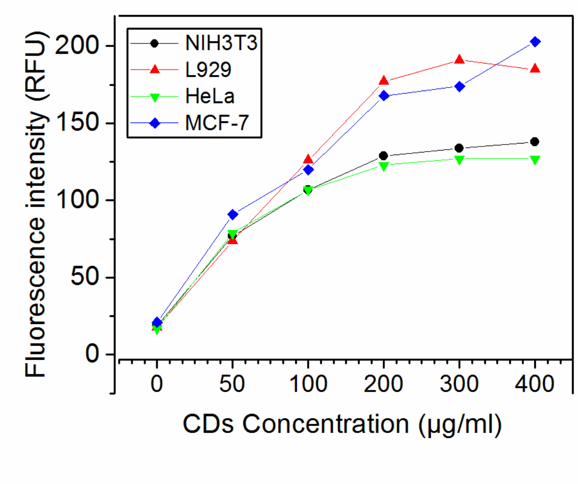

Figure S1. Concentration dependent uptake—comparison of cancer cells with mouse fibroblasts.
